# Care of the Teeth

**Published:** 1862-07

**Authors:** J. Taft


### CARE OF THE TEETH.

BY J. TAFT.

THE question is often asked, "How shall I take care of my teeth?" "What is the best method to preserve my teeth?" These and similar queries are often propounded. To answer these and such inquiries in a plain, comprehensive and understandable manner is the object of these suggestions. Various motives prompt these and like queries. They are sometimes to gratify an idle curiosity; others as a kind of excuse for past neglect; others from a real desire to attain knowledge which shall be for their own advantage, and which they intend faithfully to put in practice; it is for the latter class we now write. The precise course that is indicated may be modified and governed by many circumstances, and these exceedingly diverse. The character and organization of the teeth must always be taken into consideration. This, of course, involves their original formation and organization, whether they have strong or feeble vitality. This is usually governed by that of the general system. The condition of the parts about the teeth must be regarded, whether in a healthy or diseased condition, or predisposed to disease, and if so, whether it depends upon local or general causes. It will be impossible to keep the teeth in a proper condition while surrounding tissues are in a diseased state.

It will also be important to know the condition of the general system. Though the teeth may not always be materially injured by a general affection further than is induced by an abnormal condition of the vitality; yet there are affections under whose influence the teeth are cut down with fearful rapidity. Anything that will materially change the secretions, and especially that of the mucous membrane, will injuriously affect the teeth. Indigestion or dyspepsia usually works injury to the teeth.

We might enumerate many things of this kind, but to the intelligent it is unnecessary, and those who are not intelligent upon these subjects we do not intend here to tie down to a few specifications; they should study principles.

Those persons to whom we will first make suggestions are those of a good, healthy organization; the teeth free from disease, or comparatively so. To such we would say first, maintain as nearly perfect health as possible, in every particular; never permit anything to come in contact with the teeth that will be injurious to them. Some things, as acids for example, will act upon them chemically; again, mechanical injury may be done to the teeth by biting on hard substances, or by striking hard blows upon them, or wrenching them; excessive variations of temperature should be avoided —at least those that will produce pain or unpleasant sensations.

The teeth should be kept perfectly free from all deposits. These are from two sources, viz., from the saliva, and from the lodgment of food upon and between the teeth. Usually, where there is good health, there is no deposit from the saliva, though there are some very marked exceptions, but the general rule is as we have indicated. In all cases where this deposit is made, it should be removed. The method of doing this will be determined by its character; if it is soft, it may by close attention be always removed with the brush alone, or if not thus, with the aid of some suitable dentifrice that will act *mechanically* upon it, but never *chemically*, that is, to dissolve it. If the brush and dentifrice will not perfectly remove it, then an instrument must be resorted to, either by the person himself, or by some one competent to use it. There is a prejudice against the use of an instrument of metal upon the teeth, in the hands of the patient at least; to such an extent does this exist, that persons often attribute the decay of all the teeth to the occasional presence of pins in the mouth, or the occasional use of a pin as a tooth-pick. But no injury can be done to the teeth by the judicious use of instruments for removing deposits from the teeth. After the instruments have been used, however, upon the teeth, the part should be well polished with a brush and dentifrice, or with soft wood instead of the brush.

This deposit—commonly called tartar—does not produce decay of the teeth direct, but brings about a vitiated state of the mouth, which is very prejudicial to the teeth; it also, in many cases, produces absorption of the gums and processes, thus occasioning the ultimate loss of the teeth.

Those things that produce the great proportion of decay of teeth are the deposits from food, or portions of the food and like substances remaining upon and between the teeth till it becomes vitiated, and then acts upon the tooth bone. This can always be removed with the brush and suitable dentifrices, and sufficient effort should be exercised to accomplish it.

It should be a universal rule to keep the teeth free from all deposits or lodgment of foreign substances whatever. This can not be done always with the brush alone, but picks are required for the removal of substances from between the teeth; floss silk or tape should also be used for polishing the proximal surfaces.

(To be continued.)

